# Proteasome modulator 9 and macrovascular pathology of T2D

**DOI:** 10.1186/1475-2840-10-32

**Published:** 2011-04-17

**Authors:** Claudia Gragnoli

**Affiliations:** 1 Laboratory of Molecular Genetics of Complex and Monogenic Disorders, Department of Medicine and Cellular & Molecular Physiology and Biostatistics, Penn State University and M. S. Hershey Medical Center, Hershey, PA, USA; 2 Center for Biotechnology and Department of Biology, Temple University's College of Science & Technology, Philadelphia, PA, USA; 3 Molecular Biology Laboratory, Bios Biotech Multi-Diagnostic Health Center, Rome, Italy

## Abstract

**Aims:**

Coronary artery disease (CAD) and stroke share a major linkage at the chromosome 12q24 locus. The same chromosome region entails at least a major risk gene for type 2 diabetes (T2D) within NIDDM2, the non-insulin-dependent-diabetes 2 locus. The gene of Proteasome Modulator 9 (*PSMD9) *lies in the NIDDM2 region and is implicated in diabetes in mice. *PSMD9 *mutations rarely cause T2D and common variants are linked to both late-onset T2D and maturity-onset-diabetes of the young (MODY3). In this study, we aimed at determining whether *PSMD9 *is linked to macrovascular pathology of T2D.

**Methods and Results:**

In our 200 T2D families from Italy, we characterized the clinical phenotype of macrovascular pathology by defining the subjects for presence or absence of CAD, stroke and/or transitory ischemic attacks (TIA), plaques of the large arterial vessels (macro-vasculopathy) and arterial angioplasty performance. We then screened 200 T2D siblings/families for *PSMD9 +nt460A/G*, *+nt437C/T *and exon *E197G A/G *single nucleotide polymorphisms *(*SNPs) and performed a non-parametric linkage study to test for linkage for coronary artery disease, stroke/TIA, macro-vasculopathy and macrovascular pathology of T2D. We performed 1,000 replicates to test the power of our significant results. Our results show a consistent significant LOD score in linkage with all the above-mentioned phenotypes. Our 1000 simulation analyses, performed for each single test, confirm that the results are not due to random chance.

**Conclusions:**

In summary, the *PSMD9 IVS3+nt460A/G*, *+nt437C/T *and exon *E197G A/G SNPs *are linked to CAD, stroke/TIA and macrovascular pathology of T2D in Italians.

## Introduction

Type 2 diabetes (T2D) has long-term complications, both microvascular and macrovascular. The microvascular complications entail the diabetic retinopathy, diabetic neuropathy and diabetic nephropathy. The macrovascular complications are represented by coronary artery disease (CAD), stroke, transitory ischemic attacks (TIA), and atherosclerotic plaques in major arterial vessels. The history of arterial angioplasty in a patient also implicates the presence of macro-vasculopathy. Given the high impact of macrovascular pathology on life quality and life expectancy, it is quite important to identify risk genes contributing to such polygenic and complex phenotype.

Recently, a study reported a significant linkage to coronary artery disease (CAD) [1], myocardial infarction [1,2], stroke [3] to the chromosomal locus 12q24, within the NIDDM2 locus in which *PSMD9 *is located.

*PSMD9 *unique mutations rarely cause T2D [4]. We identified a significant linkage of *PSMD9 *common single nucleotide polymorphism (SNPs) to late-onset T2D, via recessive/additive model [5], as well as to early-onset T2D/MODY3 via additive model in Italians families [6]. The PSMD9 role in beta cell dysfunction and T2D in mice is known [7].

The aim of the present study was to determine whether the *IVS3+nt460A/G*, *+nt437C/T *and exon *E197G A/G *T2D risk (SNPs) of *PSMD9 *cause evidence for linkage with other T2D-associated phenotypes such as CAD, stroke/TIA, vasculopathy and macrovascular pathology of T2D in Italians.

## Methods

### Ethical Statement

The subjects were all recruited from Rome, Italy following the Helsinki declaration guidelines. Subjects gave written informed consent and the Penn State College of Medicine Ethical Committee approved the study.

### Families

We previously recruited 200 Italian T2D affected siblings and families with both T2D affected and unaffected members [8]. We excluded all families with identical twins, both parents affected and genetic admixture with no Italians. We confirmed that the families were for at least three generations Italians. In addition, the Italians are suitable for genetic studies as they are a homogeneous population from a geographically restricted peninsula.

### Clinical Phenotyping

We characterized the family subjects with T2D and without T2D for presence/absence of coronary arterial disease (CAD: angina or myocardial infarction/coronary angioplasty/presence of pathological Q waves on the electrocardiogram), cerebrovascular events (stroke/transitory ischemic attacks), vasculopathy (plaque(s) on carotid arteries/lower extremities arteries, and/or arterial angioplasty), and macrovascular pathology (vasculopathy and/or CAD and/or cerebrovascular events). Phenotypes of macrovascular pathology are described as unknown if diagnosis is unclear or data are lacking; subjects are described as affected if they carry the phenotype under study and as unaffected if they do not have the phenotype. In our 200 Italian families, for each phenotype, data are less than 100%.

### Sequencing

We sequenced the *PSMD9 IVS3+nt460*/*+nt437/E197G *SNPs in all family members. We directly sequenced the PCR products, status post-purification via EXOSAP-IT on an automated ABI 3730 Sequencer.

### Statistical Analysis

We tested in the 200 Italian families for linkage of the *PSMD9 *SNPs with all described phenotypes. The three SNPs studied are in linkage disequilibrium as previously shown [4]. The map distance between each SNP pair given for the linkage analysis was 0.1 cM. We performed our statistical analysis using the latest version of Merlin software [9] and the allelic information of a total of 200 genotyped families. Merlin analyzed all the informative families within this dataset. Non-parametric linkage analysis was performed for the *IVS3+nt460A/G*, *+nt437C/T *and exon *E197G A/G *SNPs. Each phenotype was tested independently using for the above-mentioned three SNPs the same map distance (cm 139.1, 139.2, 139.3 on the same chromosome, respectively). Merlin calculated the linkage score for each of these SNPs in linkage disequilibrium as a single locus, as it reported only one LOD score per statistical test, which implies that Merlin considered the SNPs located on a single locus.

All results are reported as LOD scores calculated by Merlin. For each test performed for each specific phenotype, to exclude false positive results, we calculated the empirical P-value in 1,000 replicates of simulations by using the gene dropping method: this analysis replaces real data with simulated data, while maintaining the pedigree structure, allele frequencies and recombination fraction. These datasets are generated under the null hypothesis of no linkage. The empirical P-value for each phenotype-specific test was calculated based on the P-value corresponding to the single LOD score given by Merlin for the three SNPs analysis at one locus and using the same map distance of the real analysis. Further, the outcome of the simulations was calculated taking in consideration how many times in 1,000 replicates there was a P-value equal to or smaller than the P-value of the real analysis.

## Results

The LOD scores of the non-parametric linkage analysis, performed for the various phenotypes, are reported, jointly for the three tested SNPs, with their corresponding P-value as well as with the results of the 1000 simulations (Table 1). Linkage is shown significantly for the CAD phenotype, the stroke/TIA phenotype and the macrovascular pathology phenotype and with a less significant empirical P-value with the vasculopathy phenotype.

**Table 1:** Non-parametric Linkage Analysis of Various Phenotypes of the 200 Italian Families by Merlin software.

Phenotype	Prevalence	Families	Lod Score	P-value	Empirical P-value
**CAD**	28.20%	15	1.39	0.006	0.005
**Stroke**	14.80%	5	1.04	0.014	0.002
**Vasculopathy**	53.80%	48	0.64	0.040	0.053
**Macrovascular Pathology**	70.10%	78	2.21	0.0007	0.002

Prevalence = phenotype prevalence among the family subjects studied; Families = families number analyzed; Lod score = derived from the non-parametric linkage analysis by Merlin; Empirical P-value = derived from 1000 replicates by using the gene dropping method; CAD = coronary artery disease; Vasculopathy = based on the presence of plaques and/or angioplasty of carotid arteries, coronary arteries, lower extremities arteries; Macrovascular Pathology = based on the presence of CAD and/or Stroke/TIA and/or Vasculopathy.

## Discussion

Our study shows that the *PSMD9 IVS3+nt460A/G*, *+nt437C/T *and exon *E197G A/G *SNPs studied are in linkage with CAD, cerebrovascular events, vasculopathy, and macrovascular pathology. The simulation analyses rule out false results for the linkage to CAD, stroke and macrovascular pathology at the significance level of at least α = 0.005, however the significance level of the vasculopathy linkage is only at α = 0.053. Thus, *PSMD9 *or any of its variants contribute to linkage of the reported phenotypes.

Of note, the potential contribution of intronic variants to complex disorders such as atherosclerosis and/or major cardiovascular events is widely accepted.

However, we think it is important to take in consideration that the linkage signal detected for the macrovascular pathology phenotypes in our study may indeed reflect the underlying linkage for T2D [5]. In fact, we recognize that it is difficult to disentangle associated complex phenotypes in a family-based linkage study targeted at one phenotype only at a time. Despite the necessity to raise concerns about the potential implications of the statistical data, we think that it is possible that a gene responsible for T2D is also contributing to macrovascular disease. This interpretation is based on the fact that the two complex phenotypes are strongly associated as well as that the same locus is linked in multiple studies to T2D [10,11] and macrovascular disease [3,1].

*PSMD9 *gene has the potential of having pleiotropic effects. Recently, we published the contribution of the same gene to T2D-nephropathy [12], which is a microvascular complication of T2D and is linked to the 12q24 locus [13].

Our results should be replicated in different T2D ethnic groups and are of relevance to the T2D families as well as to the families with macrovascular pathology without T2D carrying linkage in the chromosome 12q24 locus, which may be explained by the *PSMD9 *variants, at least partially. This locus in fact was reported as linked to macrovascular pathology in other populations [1-3]. In addition, we hereby prove the strength and validity of the SNP-based linkage approach in identifying genes in complex disorders. Microsatellite used mostly in the past as markers for linkage studies in polygenic diseases should be revisited, as they may have disempowered the linkage strategy. Also, family-based linkage studies are stronger in their statistical power compared to association studies. Our Italian family dataset is ethnically homogenous, thus it allows significant results to be revealed. The mechanism by which the PSMD9 protein may contribute to the macrovascular pathology is unknown at the present time.

Lifetime expectancy is highly affected by the presence of atherosclerosis and macrovascular pathology, thus the impact of the identification of a risk gene to macrovascular pathology is inestimable.

## Conflict of interests

The authors declare that they have no competing interests.

## Authors' contributions

CG conceived and designed the study, collected the clinical information, performed the statistical analysis and drafted the manuscript.

## References

[B1] Zintzaras E Kitsios G Identification of chromosomal regions linked to premature myocardial infarction: a meta-analysis of whole-genome searches J Hum Genet 2006 51 1015 10.1007/s10038-006-0053-x 17024316

[B2] Gudbjartsson DF Bjornsdottir US Halapi E Helgadottir A Sulem P Jonsdottir GM Thorleifsson G Helgadottir H Steinthorsdottir V Stefansson H Williams C Hui J Beilby J Warrington NM James A Palmer LJ Koppelman GH Heinzmann A Krueger M Boezen HM Wheatley A Altmuller J Shin HD Uh ST Cheong HS Jonsdottir B Gislason D Park CS Rasmussen LM Porsbjerg C Hansen JW Backer V Werge T Janson C Jonsson UB Ng MC Chan J So WY Ma R Shah SH Granger CB Quyyumi AA Levey AI Vaccarino V Reilly MP Rader DJ Williams MJ van Rij AM Jones GT Trabetti E Malerba G Pignatti PF Boner A Pescollderungg L Girelli D Olivieri O Martinelli N Ludviksson BR Ludviksdottir D Eyjolfsson GI Arnar D Thorgeirsson G Deichmann K Thompson PJ Wjst M Hall IP Postma DS Gislason T Gulcher J Kong A Jonsdottir I Thorsteinsdottir U Stefansson K Sequence variants affecting eosinophil numbers associate with asthma and myocardial infarction Nat Genet 2009 41 342 10.1038/ng.323 19198610

[B3] Sherva R Miller MB Pankow JS Hunt SC Boerwinkle E Mosley TH Weder AB Curb JD Luke A Morrison AC Fornage M Arnett DK A whole-genome scan for stroke or myocardial infarction in family blood pressure program families Stroke 2008 39 1115 10.1161/STROKEAHA.107.490433 18323513

[B4] Gragnoli C Cronsell J PSMD9 gene variants within NIDDM2 may rarely contribute to type 2 diabetes J Cell Physiol 2007 212 568 10.1002/jcp.21127 17516568

[B5] Gragnoli C PSMD9 gene in the NIDDM2 locus is linked to type 2 diabetes in Italians J Cell Physiol 2010 222 265 10.1002/jcp.21954 19877155

[B6] Gragnoli C PSMD9 is linked to MODY3 J Cell Physiol 2010 223 1 20069546 10.1002/jcp.22007

[B7] Thomas MKYK Tenser MS Wong GG Habener JF Bridge-1, a novel PDZ-domain coactivator of E2A-mediated regulation of insulin gene transcription Mol Cell Biol 1999 19 8492 10567574 10.1128/mcb.19.12.8492 PMC84960

[B8] Milord E Gragnoli C NEUROG3 variants and type 2 diabetes in Italians Minerva Med 2006 97 373 17146417

[B9] Abecasis GR Cherny SS Cookson WO Cardon LR Merlin--rapid analysis of dense genetic maps using sparse gene flow trees Nat Genet 2002 30 97 10.1038/ng786 11731797

[B10] Mahtani MM Widen E Lehto M Thomas J McCarthy M Brayer J Bryant B Chan G Daly M Forsblom C Kanninen T Kirby A Kruglyak L Munnelly K Parkkonen M Reeve-Daly MP Weaver A Brettin T Duyk G Lander ES Groop LC Mapping of a gene for type 2 diabetes associated with an insulin secretion defect by a genome scan in Finnish families Nat Genet 1996 14 90 10.1038/ng0996-90 8782826

[B11] Wiltshire SFT Groves CJ Levy JC Hitman GA Sampson M Walker M Menzel S Hattersley AT Cardon LRMM Evidence from a large U.K. family collection that genes influencing age of onset of type 2 diabetes map to chromosome 12p and to the MODY3/NIDDM2 locus on 12q24 Diabetes 2004 53 855 10.2337/diabetes.53.3.855 14988275

[B12] Gragnoli C T2D-Nephropathy Linkage within 12q24 Locus Diabetes Res Clin Pract 2011 in press 10.1016/j.diabres.2011.02.026 21439668

[B13] Bowden DW Sale M Howard TD Qadri A Spray BJ Rothschild CB Akots G Rich SS Freedman BI Linkage of genetic markers on human chromosomes 20 and 12 to NIDDM in Caucasian sib pairs with a history of diabetic nephropathy Diabetes 1997 46 882 10.2337/diabetes.46.5.882 9133559

